# Extreme Rare Events Identification Through Jaynes Inferential Approach

**DOI:** 10.1089/big.2021.0191

**Published:** 2021-12-10

**Authors:** Yair Neuman, Yochai Cohen, Eden Erez

**Affiliations:** ^1^ The Department of Cognitive and Brain Sciences, The Zlotowski Center for Neuroscience, and The Data Science Research Center, Ben-Gurion University of the Negev, Beer-Sheva, Israel.; ^2^ Gilasio Coding, Tel-Aviv, Israel.; ^3^ Independent Researcher, Tel-Aviv, Israel.

**Keywords:** extreme rare events, feature engineering, inference, Jaynes, pulp-and-paper

## Abstract

The identification of extreme rare events is a challenge that appears in several real-world contexts, from screening for solo perpetrators to the prediction of failures in industrial production. In this article, we explain the challenge and present a new methodology for addressing it, a methodology that may be considered in terms of features engineering. This methodology, which is based on Jaynes inferential approach, is tested on a dataset dealing with failures in production in the pulp-and-paper industry. The results are discussed in the context of the benefits of using the approach for features engineering in practical contexts involving measurable risks.

## Introduction

The identification, whether diagnosis, prediction, or classification, of extreme rare events may be presented, discussed, and explained by considering it in terms of “finding a needle in a haystack.” The idiom “a needle in a haystack” is used to describe an object that is very difficult or even impossible to locate. The *Oxford English Dictionary* further explains that the object is difficult or impossible to find because “it is hidden among so many other things.”^1^ These “things” in which the object is hidden are similar to the object to a degree where they cannot easily be distinguished. For instance, finding a needle in a box containing numerous particles of black granular material may be less difficult than finding a bamboo knitting needle in a stack of bamboo shoots. In other words, a low prevalence of an event is an insufficient condition for defining it as a “needle in a haystack” as a “needle” may have clear distinguishing features. Therefore, finding a needle in a haystack is different from the context of anomaly detection where “*by definition*” the anomaly has clear distinguishing features.

“Anomaly detection is the process of identifying unexpected items or events in datasets, which differ from the norm.”^2^ Finding a needle in a haystack is clearly not a context where items or events differ from a norm in the trivial sense of having simple distinguishing features.^3^ For example, some rare events, such as credit frauds,^4^ can be successfully identified through machine learning (ML) algorithms because they have a strong distinguishing signature regardless of their rarity. The difficulty of finding a needle in a haystack is, therefore, associated not only with the *number* of things in which the object is hidden but also with the *quality* of the things that cannot be easily distinguished from the object (i.e., the needle). This challenging context appears, for instance, in screening for solo perpetrators (e.g., Refs.^3,5^), where it is far from trivial to find a distinguishing signature of a perpetrator in a high-dimensional space of features characterizing an individual.

Moreover, the challenge of finding a needle in a haystack may be accompanied by the rate of false positives (FPs) or “false alarms” (for an excellent discussion see Ref.^6^). In their paper “Visual Reflections: Until Proven Guilty,” Wainer and Savage^7^ explain the problem in the context of screening for potential terrorists. The low prevalence of terrorists, or solo perpetrators in general, is such that even a highly accurate diagnostic test is prone to producing false alarms (i.e., FPs) to a degree that might make the test irrelevant for all practical purposes. Therefore, in practical real-world contexts, which are the focus of this article, the needle in the haystack problem is entangled with the cost of FPs. It is a context characterized by:

(1) a phenomenon with low prevalence
(2) lacking a few simple distinguishable features and
(3) accompanied by the cost of FPs *regardless* of the test diagnostic performance.

Appendix A1 includes an elaborated example illustrating the problem of FPs in the context of diagnosing a rare personality disorder.

## Identifying Rare Events Through the Bayesian Approach

The engineering of features for the identification of extreme rare events may benefit from a Bayesian approach. We explain this idea by using the concept of “odds.” In contrast with probability, odds concern the ratio of positive to negative cases. In this context, the identification of extreme rare events may be performed by updating our prior odds of an extreme rare event using the diagnostic value of a signal. Here, we may use the odds of gaining a positive test result or a signal (i.e., the evidence, *E*) given the hypothesis (i.e., *H* = extreme rare event) versus gaining a positive test result given the complementary hypothesis (-extreme rare event):

$$\frac{p\left(E∕H\right)}{p\left(E∕-H\right)}$$

This ratio is known as the Bayes factor (BF).^8^ By multiplying the prior odds for a given hypothesis by the BF, we get the posterior odds in favor of the hypothesis:

$$\frac{p\left(H∕E\right)}{P\left(-H∕E\right)}=\frac{O\left(H\right)}{O\left(-H\right)}\times \frac{p\left(E∕H\right)}{P\left(E∕-H\right)}$$

Appendix A1 illustrates the use of this approach for the diagnosis of a personality disorder. It is important to understand, that by adopting this approach, we consider the appearance of an extreme rare event as the hypothesis *H* and the prediction of an extreme rare event is performed by computing the posterior odds of the event. In this context, predicting the approaching event is a process of hypothesis testing. We elaborate this idea in the next section through Jaynes inferential approach. We then explain how this approach may be used for engineering a single feature to be fed into a ML model that aims at predicting an extreme rare event.

## Jaynes' Approach to Hypothesis Testing

Jaynes^9^ proposed measuring the weight of evidence in favor of a hypothesis (*H*) by translating the odds-based equation that appears above into a *decibel* system, where the prior evidence [i.e., *e*(*H*)] for hypothesis *H* (e.g., a rare event) is:

$$e\left(H\right)=10log_{10}\left[\frac{p\left(H\right)}{p\left(-H\right)}\right]$$

and the posterior degree of belief in *H* given the evidence *E* is:

$$e\left(H|E\right)=e\left(H\right)+10\times log_{10}\left[\frac{p\left(E|H\right)}{p\left(E|-H\right)}\right]$$

When the process involves several pieces of evidence, this produces a score that we describe as *JaynesH*:

$$JaynesH=e\left(H|E\right)=e\left(H\right)+10\sum_{i=1}^{N}\log_{10}\left[\frac{p\left(Ei|H\right)}{p\left(Ei|-H\right)}\right]$$

The use of a decibel system is expressed through the common logarithm with base 10. This means that we convert the odds by using a logarithmic transformation and multiply the result by 10. Let us assume that *p*(*E*/*H*) = *p*(*E*/−*H*). In this case, the ratio is 1, the base 10 logarithm is 0, and, therefore, the result is 0; this changes nothing about our prior belief. Now let us assume that the ratio *p*(*E*|*H*)/*P*(*E*|−*H*) = 10. In this case, increasing the odds by a factor of 10 would result in a base 10 logarithm of 1 and a Jaynes score of 10. Increasing the odds to 100 would make the base 10 logarithm 2 and the Jaynes score of 20. Increasing the odds by a factor of 10 again would result in a ratio of 1000, a base 10 logarithm of 3, and a score of 30. An increase by an order of magnitude would result in a linear increase of the base 10 logarithm and differences of 10 points in the Jaynes score.

Thus far, we have learned about the problem of finding a needle in a haystack, explained the problem in diagnostic terms, presented the odds-based interpretation of a test for finding a needle in terms of odds and the updating of earlier posterior beliefs, and elaborated on the hypothesis testing procedure (i.e., is it a needle?) in terms of Jaynes' proposal.

Jaynes' approach has distinct benefits, as it integrates several pieces of evidence into a single interpretable score. The pieces of evidence, formulated through the BF, provide a robust approach for updating a prior belief. The final Jaynes score may, therefore, be helpful in contexts where there are benefits to (1) reducing the number of features, (2) where interpretability is required, and where (3) the process does not require a simple diagnostic answer but rather the updating of prior beliefs, which is accompanied by a stepwise process of examination, such as the case where a suspicious object is prioritized in a second in-depth inspection after some worrying warning signs have been identified. For example, Neuman et al.^3^ used the approach to identify psychopathic texts and Neuman et al.^10^ used it to identify potential school shooters. The aim of the current article is to illustrate the benefits of using Jaynes approach for extreme rare event identification, specifically for the engineering of a single diagnostic feature/variable aims to predict the extreme event. The next section further illustrates the Jaynes approach to extreme rare event identification, specifically in the context of predicting failures in production.

## Materials and Methods

### The dataset

The dataset we used^11^ deals with the classification of rare events in the pulp-and-paper manufacturing context. The data were taken from a paper manufacturing machine, where paper breakage is a rare but highly costly event. The prevalence of paper breakage is low: only 124 cases out of 18,398 records, which as a rounded percentage is <1% (0.7%). The dataset also includes predictor variables (*x*1–*x*61) gathered from the sensors of the machine. The challenge addressed by the authors^11^ was to predict a failure in production given the sensors' measurements at previous time points. In other words, the task was to predict a failure in advance. By training two ML classifiers (XGBoost and AdaBoost) on 0.9 of the data and testing the classifiers on the rest, the authors^11^ gained an *f*1 score of 0.114, a precision of 7%, and a false positive rate (FPR) of 2.6%.

A failure entails that production must be stopped at a cost of $10,000 per hour (C. Ranjan, pers. comm.). Over the period covered by the dataset (around a month), the loss caused by the machine failures was $1,240,000. What is the cost of a “false alarm” (a FP)? Around 475 cases were FPs. Let us assume that the cost of checking and verifying that there is no problem is 10 minutes of work at a cost of $1667. In this case, the overall cost of the FPs during the period covered by the dataset would be $791,825. If it took 15 minutes to check whether there was a problem, the cost of the FPs would be $1,187,500. What we can see is that the cost of an FP is such that any small reduction or increase may have significant financial consequences for production.

As argued by Savage,^12^ it is not uncertainty *per se* that we wish to resolve but the risk accompanying it. As risk involves cost and is “in the eye of the beholder”^12^ (p. 154), Savage proposes adopting a “risk attitude” rather than an unrealistic form of utility theory and a decontextualized attitude where uncertainly is measured in a purely academic context regardless of real-world costs and benefits. In the following section, we follow Savage in describing the costs accompanying the use of various ML classifiers to predict an approaching failure in the machine. We then test Jaynes inferential approach and show its benefits.

### Preprocessing

We used the 60 continuous features in the dataset excluding the binary variable from the analysis. For each continuous variable, we defined three measures of change. In other words, we defined three measures of change for each sensor's measurement. The first measure was:

(1) $$\delta_{xiratio}=\frac{xit}{xi_{t}-1}$$

The second measure is simply the ratio between the measurement at a time point and the measurement at the previous time point:

(2) $$\delta_{xiratio2}=\frac{1}{2}\left(\frac{xit}{xi_{t}-1}+\frac{xit-1}{{xi}_{{}_{}t}-2}\right)e$$

The third measure is:

(3) $$\delta_{xiratioratio}=\frac{\delta xiratio}{\delta xiratio2}$$

This procedure resulted in 180 new features. In the following sections, we present two rounds of analysis in an increasing order of complexity.

## Analysis 1: The Diagnostic Utility of ML Models

Recalling Savage,^12^ we cannot use the classifiers' performance measures *per se* but must evaluate them in the practical context of risk and decision making. In this case, the FP is a crucial measure. Given the cost saved through a true positives (TP) (i.e., hit or identifying the failure in advance), which is $10,000, the cost of missing a failure (i.e., false negatives [FN]), which is the same, and the estimated cost of a false alarm (i.e., FP), which is 5 minutes of work (∼$833), we can easily compute the benefit of a classifier through the following equation:

$$MONEYSAVED=TP\times COST_{TP}-FP\times COST_{FP}$$

or, in terms of hours of work saved (HWS):

HWS=TP−FN∕12

where an hour of work is priced at $10,000. To illustrate the importance of the above two measures (i.e., HWS and MONEY SAVED), consider the following analysis. As the dataset is imbalanced, we used an ML classifier with a weighted training procedure and the scaling of the features.^13^ We used the Linear SVM classifier and gained 60% recall and 0.6% precision. Given a TP of *N* = 75, we correctly anticipated a failure and stopped the machine 75 times at a cost of $750,000 instead of $1,240,000. This is clearly a significant saving in production costs. However, this classifier produced *N* = 12,801 FPs, where production must be stopped, and the machine must be checked despite the fact that no real problem exists. Even if we assume that it takes only 1 minute of work to verify that an FP is not a real failure in production, then the cost of the FPs (i.e., $2,124,966) would be such to make the use of the classifier irrelevant for all practical purposes.

The use of ML to detect extreme rare events may be accompanied by several problems that cannot be addressed even by the weighted training methodology that we applied to handle the imbalanced dataset. First, the cross-validation method may be biased regarding imbalanced sets^14^ because the distribution of cases is skewed and might distort the representation of the minority class in the folds. The way to address this difficulty is to apply *stratified k-fold cross-validation*. Therefore, we re-analyzed the data by using a stratified 10-fold cross-validation procedure with weighted training and scaling of the features. Some classifiers (such as Decision Tree, Linear SVM and GaussianNB) failed to produce any significant results, showing how sensitive an ML model is to various specifications. The results of the top three classifiers are presented in Table 1.

**Table 1.** Performance of the three best classifiers

Classifier	Recall	Precision	HWS
Gradient Boosting	55%	19%	44
AdaBoost	57%	15%	39
Random Forest	64%	12%	29

The baseline for comparison is 124 cases of failure with an accompanying cost of $1,240,000.

HWS, hours of work saved.

Let us examine the performance of the first classifier (Gradient Boosting). It produced *TP* = 68, which means that using the classifier enables us to predict 68 cases of failure in advance. Therefore, instead of working for 124 hours to correct undetectable failures, we could have worked for only 56 hours (with the remaining 56 hours resulting from our inability to correctly identify 56 of the failures in advance). We will now theoretically spend only $680,000 for the 68 working hours of fixing the machine instead of $1,240,000. However, this classifier also produced *FP* = 286. This means that we have 286 cases of false alarms where we need to stop the machine although there is nothing wrong with it. If we assume that the minimum time for verifying a false alarm is 5 minutes, then given the cost of an hour of work, working for 5 minutes should cost $833 (rounded) and the cost of 286 false alarms would be $238,238. Therefore, $441,762 (i.e., $680,000–$238,238) is the actual amount of money that we could have saved by applying the classifier. This sum is equal to 44 working hours (rounded) saved on fixing the machine.

We can see that all three classifiers produced significant results in terms of HWS. In the best-case scenario, 35% of the working hours (44/124) would be saved by using the classifier to anticipate failures. However, the use of the classifiers may be accompanied by problems. For example, in a high-dimensional imbalanced dataset, the ratio between features and observations may pose a problem and various methodologies have been designed to solve this problem (e.g., Ref.^15^). In the next section, we present the use of a single score, which is based on Jaynes approach, as the only feature in the classification task.

## Analysis 2: Using Jaynes' Approach for Engineering a Single Feature

### Identifying the cut-off value of the features

Up to now, we have used features that we have engineered. In this section, we experiment with using Jaynes' approach. To use Jaynes' approach, we must calculate the BF for each variable, and this requires a cut-off value to convert each variable into a binary form. There are several approaches to identifying an optimal cut-off value in diagnostic tests (e.g., Ref.^16^). However, for the current study, we used the following heuristics. We used *optimal binning*^17^ with respect to the dependent variable (*y*) and class (1), which is indicative of a failure in the machine. Optimal binning is a supervised method for discretizing a variable. The method we used applies the *minimum description length principle*, where cut-off values are chosen to minimize the entropy of the resulting bins. Only features that had been successfully discretized using the optimal binning procedure were selected for the next phase and entered *receiver operating characteristic (ROC) analysis*. Following the ROC analysis, features only entered the next step when the lower bound of their area under the curve (95% confidence interval) scored higher than 0.50. For each of the seven features/variables selected through this procedure, we identified the cut-off value that maximized their diagnostic ability. The pseudo-code for this procedure is as follows:

cut_off_values = list[N]
For feature index i from 1 to N
binned = Apply Optimal binning for Feature-i
If Feature-i is binned then
lower-bound = apply ROC analysis to Feature-i,
If lower-bound score >0.50 then
select the feature and identify the cut-off value maximizing diagnostic ability
cut_off_values[i] = cut-off value
otherwise remove the feature from the list
Return cut_off_values

### Computing the BF

Next, we used the following procedure. For each variable, we ran 100 iterations in which 50 cases of *y* = 1 (i.e., a failure in production) and 10,000 of *y* = 0 were randomly selected from the whole dataset. If the feature scored equal to or higher than the identified cut-off value of the selected variable/feature, it was converted to 1; otherwise, it was converted to 0. The BF of each relevant feature was calculated for each run and averaged across the 100 runs. Using this heuristic, we identified seven features whose BF was higher than 2.5, which was the arbitrary value that we chose as a cut-off point. These seven features then progressed to the next steps. The features appear in Appendix A2. The pseudo-code for computing the BF of a selected feature is as follows:

Input: cut_off_values
BF_values = array[100][N]
For *t* = 1 to 100 (100 runs)
current_set = Randomly sample 50 cases in which *y* = 1 and 10,000 cases in which *y* = 0
For feature's index *i* = 1 to N
For each case in the current_set
IF case[i] > cut_off_value[i]
Case[i] = 1
Otherwise case[i] = 0
BF_values[t][i] = Compute the BF of Feature-i
For feature's index *i* = 1 to N
Average the BF across the 100 runs (BF_values[][i]) to produce BFxi

### Measuring the Jaynes score

A new set (Set 2) of 50 cases where *y* = 1 and 10,000 cases where *y* = 0 was randomly sampled from the dataset. In this dataset, the prevalence of failures was extremely low (*p* = 0.005% or 0.5%). Only the seven variables previously identified were used to calculate the *JaynesH* score, where a variable that scored equal to or above its cut-off value was replaced by the appropriate *BF_xi_* (previously computed). The Jaynes score for this set was computed as follows:

$$JaynesH=\left(-23\right)+10\sum_{i=1}^{7}\log_{10}BFxi$$

where the prior odds in favor of the hypothesis that we are anticipating a failure are −23.

The general procedure for computing the JaynesH score is:

1. Given a dataset T
2. Compute the prior odds for the extreme event:
  $$e\left(H\right)=10log_{10}\left[\frac{probability\left(y=1\right)}{probability\left(y=0\right)}\right]$$
3. Use the BF, computed for each of the selected features, for computing the JaynesH score:

$$JaynesH=e\left(H\right)+10\sum_{i=1}^{N}\log_{10}BFxi$$

How diagnostic is the *JaynesH*? To answer this question, we simply ranked the cases in descending order according to their *JaynesH* scores. As only a Jaynes score higher than 1 is relevant, we used this cut-off value and counted the number of cases where *y* = 1 was found among these top-ranked cases. Out of 124 cases in which *JaynesH* >1, 19 were tagged as failures (i.e., *y* = 1). The result was that *TP* = 19 and *FP* = 105, and both precision and recall were 15%. By using this simple ranking approach, 10 hours of work (i.e., $100,000) could be saved, even without applying any ML model.

**FIG. 1.**
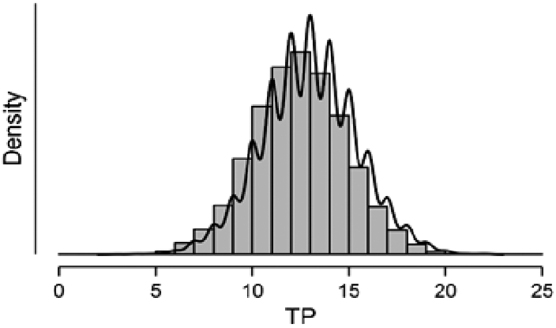
The distribution of TP. TP, true positives.

A different heuristic may be as follows. As we expect 50 cases to be tagged as 1 (i.e., a failure in production), we may simply check the number of failures among the 50 top-rated cases. In this scenario, we found that 17 cases (34%) were failures and that 14 hours of work (i.e., $140,000) could have been saved by using this simple ranking procedure. To test the reliability of these results, we ran a bootstrapping procedure where for 10,000 runs, 50 cases of *y* = 1 and 10,000 cases of *y* = 0 were randomly sampled. For each run, we computed the *JaynesH* scores, ranked the results in descending order, and counted the number of TPs in the top 50 cases. The distribution appears in Figure 1.

We found that this bell-shaped distribution had a mean of 12.94 (*Sd* = 2.4). Rounding the results, our simple ranking methodology could save on average 10 hours of work (i.e., $100,000) with a range between 8 and 12. This result suggests that computing a cut-off value for the Jaynes score may be a simple and helpful heuristic for identifying a failure in advance. Although this result cannot compete with those gained through the ML algorithms, it is surprisingly helpful and simpler as it does not rely on the use of any ML classifier. Next, we randomly produced a new set of 50 cases of *y* = 1 and 10,000 of *y* = 0 (Set 3). This was our final test set. For each case in this dataset, we produced the *JaynesH* score.

## Testing the Diagnostic Value of Jaynes as a Single Feature in an ML Model

First, we applied the ranking heuristic presented earlier. By ranking the cases in descending order according to their *JaynesH* score, we found *TP* = 12 in the top 50 cases (HWS = 9) and *TP* = 19 among the top 106 cases that gained a positive *JaynesH* score (HWS = 12). Next, we used all of the 180 features with 10 ML classifiers, such as Random Forest, GaussianNB, and Linear SVM. In terms of HWS, only 4 out of 10 classifiers produced significant results. As one of these classifiers (i.e., Randomized Trees) produced only three HWS, it is not discussed here. The performance of the top three classifiers is presented in Table 2.

**Table 2.** Performance of the top three classifiers

Classifier	Recall	Precision	TP	FP	HWS
Random Forest	55%	19%	18	65	13
AdaBoost Tree	57%	15%	14	47	10
Gradient Boosting	64%	12%	17	95	9

All results are rounded.

FP, false positive; TP, true positive.

Using the whole set of features, the best classifier applied to this set (Random Forest) achieved 13 HWS, which is a 26% improvement in HWS. Several features were relevant to the analysis. Using Features Importance, we counted the number of features whose score was higher than 0. For Random Forest, 111 features were scored higher than 0; for AdaBoost, the number was 69; and for Gradient Boosting, the number was 63.

What is the best diagnostic result gained by using JaynesH as a single feature? Surprisingly, a Bernoulli naive Bayes classifier using *only* the *JaynesH* score achieved 12 HWS, a result that is not far from the performance gained by the best ML classifier, that used the whole set of features. The results gained regarding this set cannot be compared with those gained from the larger set (Set 1). However, they show how engineering a single feature through Jaynes' inferential approach may produce excellent results, a point explained and discussed later.

## Discussion

Dealing with imbalanced datasets for the identification of extremely rare events, from solo perpetrators to failures in production, is a nontrivial challenge for several reasons. Although many ML approaches have been developed to address it, there cannot be a single best solution to the problem, for a very basic and specific reason: For real-world challenges, which are the focus of this article, uncertainty is irrelevant unless we take into account (1) the risk analysis associated with the challenge, (2) the fact that this risk is in “the eye of the beholder”^12^ and must be resolved in a context that emphasizes updating prior beliefs, and (3) the fact that the particularities of a specific challenge necessarily invite various approaches to fine-tune the possible solutions. Using ML algorithms with their performance measures without considering the cost of false alarms, for instance, might be detrimental to the success of a project, as previously illustrated. In this context, the approach that we present in this article is fully justified.

In the context of finding a needle in a haystack, rare events may be very different from one another,^18^ forming a variety that is not trivial when optimizing an ML model. Moreover, the ratio between features and observations makes it difficult to avoid the problem of overfitting. In addition, in the practical context in which we are interested, it is highly important to produce interpretable and actionable results. For example, in the context of pulp-and-paper production, it is crucial to identify the exact source of failure to fix the problem and return to production as soon as possible. Using 180 features in an ML model makes it quite difficult to identify the source of a failure, especially when they are combined in a nonlinear model. In this context, using the Jaynes approach may have some benefits not only in reducing the number of features but also in identifying specific features that have a clear diagnostic value. To recall, the Jaynes measure is formed through the BF, which is a straightforward measure of the weight of evidence. Only features whose BF is higher than a certain threshold are selected for diagnosis and the updating of prior beliefs. In this way, the number of features is significantly reduced to choose only those that present clear diagnostic value that can easily be interpreted in terms of odds or likelihood ratio. In addition, the fact that the Jaynes score sums the contributions of a few features makes it easier to identify and fix the source of the problem, which is extremely difficult, if not impossible, in the context of a model that computes the nonlinear interactions between features, such as a neural network model.^19^

The integration of a few pieces of diagnostic evidence in a single score may, therefore, produce a simple way to test a hypothesis regarding a failure, in our specific context of production, or to identify a needle in the broader context in which we are interested. Moreover, the proposed method allows for online updating of features. If a new feature is added to the dataset, then the proposed method (as it is additive) can incorporate it into the Jaynes score without recomputing the effect of existing features. This aspect does not exist in typical ML algorithms, where the entire model needs to be retrained.

In sum, using Jaynes' approach may have clear benefits in reducing the number of features to a group whose selection criterion is easily interpretable and can be used for actionable nonacademic research. The approach presented in this study emerged from the real-world challenge of identifying extreme rare evets. Here, it has been successfully used to identify failures in production and the methodology may find various other real-world applications. The question as to whether the same approach may be successfully applied to other data sets of extreme rare events is an open question that can be answered only empirically. However, the pragmatic approach that we adopt in this article has no pretensions to provide a silver bullet for addressing the challenge of finding a needle in a haystack. We conceive the proposed approach as only one possible tool in the toolbox of a data scientist, and the utility of the tool is to be judged only in practice. It is this modest context of features' engineering for the diagnosis of rare events in which our article is located. Therefore, the article concludes by inviting further research into the development of this methodology in the context of real-world identification of extreme rare events.

## Abbreviations Used

BF: Bayes factor
FPs: false positives
FN: false negatives
FPR: false positive rate
HWS: hours of work saved
ML: machine learning
ROC: receiver operating characteristic
SVM: support vector machine
TN: true negatives
TP: true positives

## Appendix

### Appendix A1. Understanding FPs Through Diagnosis

In this appendix, we illustrate and explain the problem of FPs through the diagnosis of the antisocial psychopathic personality disorder, which is a rare and extreme disorder.

The antisocial psychopathic personality has three main traits: (1) manipulativeness, (2) impulsiveness, and (3) lacking empathy and remorse.^20^ Identifying or diagnosing psychopaths may be highly important in several contexts. For instance, some sensitive positions require rational individuals whom we can fully trust and who have clear moral norms. An FBI agent who may cross a line and cooperate with drug dealers is a serious insider threat that must be avoided. In such sensitive contexts, it is highly important to screen potential candidates for a psychopathic signature. For illustration, let us use a toy example. Assume that we have a short test for diagnosing psychopathic personality and that the test includes the following three items:

1. I feel empathy for human beings
2. I have never felt regret
3. I usually respond on impulse

Subjects are asked to judge how well each item describes them—or, if we do not trust them to honestly respond to the items, then an expert is asked to provide the evaluation (e.g., “the subject shows no empathy to human beings”). A person who scores above a certain threshold is tagged as a psychopath, and others are tagged as nonpsychopaths. For example, disagreeing with the first item and agreeing with the two last items might result in a psychopathic tag. Now, let us further assume that among 1000 individuals, only 1% are clinically diagnosed psychopaths. The prevalence of psychopaths is low, and diagnosing a psychopath seems to be a problem of finding a needle in a haystack.

When analyzing the diagnostic performance of the test, we may get the results shown in Appendix Table A1. To measure the accuracy of the test, we may use two measures: *sensitivity* and *specificity*. The sensitivity (or the *recall*) of the test is the number of *TP* divided by the number of TPs and *FN*. In other words, it is the ratio of cases our test has correctly identified as psychopaths to the total number of psychopaths. In our case, it is 9/10 = 0.90, which means that our test has 90% sensitivity: it has correctly identified 90% of the psychopaths as such. Specificity is the ratio of *true negatives* (*TN*)—those cases that the test has correctly diagnosed as nonpsychopaths—to the total number of nonpsychopaths. In our case, the specificity is 901/990 = 0.91. The *accuracy* of the test is defined as:

$$Accuracy=\frac{TP+TN}{TP+TN+FP+FN}$$

where *FP* is the number of false positives. In our case, the accuracy is 91%.

However, the test produces a high number of FPs (*N* = 89), a result that has various implications. To explain the implications, we may present an imaginary scenario followed by a question. Let us assume that the first author of this article has completed the test by answering the three items and has scored above the threshold. He is, therefore, tagged by the test as a psychopath. Given the high accuracy of the test, is it more likely that he is a psychopath than a nonpsychopath?

To answer this question, we may ask: What are the chances that he is *really* a psychopath? Stated differently, we need to consider the probability that the first author is a psychopath *given* the evidence provided by the diagnostic test:

$$P\left(Psychopath∕Testresult>threshold\right)=?$$

The result is 0.09, meaning that the probability of the first author being a psychopath given his test result is such that it is much more likely that he is *not* a psychopath. This counterintuitive result is sometimes difficult to understand by laypeople and scientists alike, as it looks like a paradox. How is it possible that the test is so accurate and yet that the first author of this paper has such a low probability of being a psychopath, given his *positive test result*?

Sanderson^6^ and Wainer and Savage^7^ explain the problem of FPs (i.e., false alarms) in the context of finding a needle in a haystack where the prevalence of the target (e.g., psychopaths) is extremely low. In explaining what he describes as the “paradox,” Sanderson suggests that we should think about a diagnostic test not as a simple and straightforward device for gaining a binary answer but as a process through which *prior odds are updated*. This approach is in line with that presented by Savage^12^ (p. 154), where risk is described as existing in the eye of the beholder (i.e., having a subjective component) and where whenever we are dealing with uncertainty, we should do it in the context of *updating our odds* as they are conceived from our specific risk-taking position. Sanderson's explanation of the “paradox” follows in the footsteps of Jaynes and his Bayesian approach to inference and hypothesis testing, which seems to be relevant to the approach proposed by Savage.

In contrast with probability, odds concern the *ratio* of positive to negative cases. In the example cited earlier, the prior odds of being a psychopath are 10 to 990 (i.e., 0.01% or 1%). If we have no information about a specific individual's responses to the test items, the odds of their being a psychopath are 1 to 99. We may update our prior odds by taking the test result into account. Here, we may use the odds of gaining a positive test result that the subject is a psychopath (i.e., the evidence, *E*) given the hypothesis (i.e., *H*) that the subject is a psychopath:

$$\frac{p\left(E∕H\right)}{p\left(E∕-H\right)}$$

This ratio is the BF. It expresses the ratio between the sensitivity of a test and its FPR:

$$BF=\frac{Sensitivity}{FPR}$$

which in our case is:

$$BF=\frac{p\left(E∕H\right)}{p\left(E∕-H\right)}=\frac{Sensitivity}{FPR}=\frac{0.90}{0.09}=10$$

It must be noted that the BF can be computed for “negative evidence” as well:

$$BF=\frac{p\left(-E∕H\right)}{p\left(-E∕-H\right)}$$

Which, in our case, provides the odds of observing a nonpsychopathic signature (i.e., a negative test result) given a psychopathic versus a nonpsychopathic personality. In this case, the BF is the *false negative rate* (FNR) divided by the specificity:

$$BF=\frac{FNR}{Specificity}$$

By multiplying the prior odds that a subject is a psychopath by the BF, we get the *posterior odds* in favor of the hypothesis that the subject is a psychopath:

$$\frac{p\left(H∕E\right)}{p\left(-H∕E\right)}=\frac{O\left(H\right)}{O\left(-H\right)}\times \frac{p\left(E∕H\right)}{p\left(E∕-H\right)}=0.01\times 10=0.1$$

Given this result, we can update our prior belief that the odds of the diagnosed subject being a psychopath are 1 to 100 to the updated belief, which is that the odds are 1 to 10. The focus here is on the *updating of beliefs* rather than on a simple binary answer. Although the odds in favor of the hypothesis that our subject is a psychopath are still low, they have been updated in a way that significantly increases our relative belief that he is a psychopath. In this case, we still hold the belief that the subject is a normal person. However, positively affirming that he does not feel empathy has never felt regret and that he is an impulsive person may increase our belief in the direction of psychopathy. In real-world contexts where we are dealing with risky situations, the “answer” provided by the diagnostic test is not a final and conclusive verdict. In real-world contexts, the updated belief—that our subject looks much more similar to a psychopath than we originally believed—invites further diagnostic steps. In the context of solo perpetrators, updating our beliefs in a way that increases our suspicion naturally leads to a stepwise process of in-depth inspection. Using Jaynes approach for the diagnosis cited earlier is detailed in the next section.

We illustrate the use of Jaynes' formulation by breaking our diagnostic test down into the three items. A subject is presented with the item “I feel empathy for human beings” and agrees with it. The probability of the subject agreeing with this item if they are a psychopath is low: *P*(*E*/*H*) = 0.01. However, the probability of the subject agreeing with this item if they are not a psychopath is high: *P*(*E*/−*H*) = 0.95. In contrast, the probability of the subject disagreeing with this item if they are a psychopath is 0.99 (i.e., 1–0.01) and the probability of them disagreeing with this item if they are a nonpsychopath is 0.05 (i.e., 1–0.95). In the case of a positive response (i.e., agreement with the item), the Jaynes score (*Jaynes^+^*) is:

$$Jaynes^{+}=10\times log10\left(\frac{0.01}{0.95}\right)=10\times -2=-20$$

Given that the prior odds of being a psychopath in Jaynes' decibel terms are:

$$e\left(H\right)=10\times log10\left(\frac{P\left(H\right)}{P\left(-H\right)}\right)=-20$$

our belief regarding the posterior odds that the subject is a psychopath is updated from −20 to −40. This means that, given full ignorance, the odds were against the hypothesis that the subject is a psychopath. When the subject affirmed that they feel empathy for human beings, we were able to strengthen our belief that they are not a psychopath by two orders of magnitude.

But what if the subject disagrees with the item, admitting that they do not feel empathy for human beings? In the case of a negative response, the Jaynes score (*Jaynes^−^*) is:

$$Jaynes^{-}=10\times log10\left(\frac{0.99}{0.05}\right)=10\times 1.98=+19.8$$

In this case, we update the odds in the other direction, increasing our suspicion that the subject is a psychopath. If the prior odds were −20, the posterior odds are now −20 + 19.8 = −0.02. At this point, we still do not hold the belief that the subject is a psychopath, but we have changed our belief in this direction in a significant way.

Appendix Table A2 shows the item in the leftmost column followed by four columns indicating different conditional probability scores and two columns indicating the corresponding Jaynes scores. Appendix Table A3 shows the updating process.

### Appendix A2.

The features identified for computing the *JaynesH*:

1. x2 ratio
2. x3 ratio
3. x2 ratio ratio
4. x3 ratio ratio
5. x11 ratio ratio
6. x18 ratio ratio
7. x19 ratio ratio

**Appendix Table A1.** Performance of the psychopathy test

Test	Observation	
	Psychopath	Nonpsychopath
Positive	9	89
Negative	1	901

**Appendix Table A2.** Scores for the first item

	P* (*E*/−*H)	P* (*E*/*H)	P* (−*E*/−*H)	P* (−*E*/*H)	Jaynes^+^	Jaynes^−^
Item 1	0.95	0.01	0.05	0.99	−20	+12.96

**Appendix Table A3.** Updating the prior belief

Item	Response	Updated odds
Prior	NA	−20
Item 1	Positive (i.e., agree)	−40
